# Onward to 2016

**DOI:** 10.5772/62278

**Published:** 2016-01-28

**Authors:** Shidong Jia, Antonio Chiesi, Winston Patrick Kuo

**Affiliations:** 1 Predicine Holdings Ltd, Hayward, CA, USA; 2 Exosomics Siena, Siena, Italy; 3 BioPharma Research Council, Tinton Falls, NJ, USA

As we embrace for another exciting year (2016), we are thrilled to announce JCB has been indexed in the DOAJ (Directory of Open Access Journals) (https://doaj.org/toc/1849-4544)! This was attributable to the number of quality published original research articles in 2015 with the help of our Associate Editors and Editorial Board. We published several papers covering each of the main topics within the scope of the journal from cell-free DNA, circulating tumour cells and extracellular vesicles to circulating proteins. In addition, we opened a new technology forum presenting the latest technology and published its first article entitled, “Analytical Validation and Capabilities of the Epic CTC Platform: Enrichment-Free Circulating Tumour Cell Detection and Characterization” by Shannon Werner and her colleagues.

Over the past year, we were proud to announce several ongoing developments such as the expansion of our Associate and Editorial Board with key opinion leaders in the circulating biomarker field including: Lawrence Rajendran (University of Zurich, Switzerland) as part of our Associate Editors team, and Quentin Qiang Liu (Dalian Medical University) and Shlomit Kenigsberg (CReATe Fertility Centre, Canada) as members of our Editorial Board.

JCB is now media partners with both SelectBio and the Cambridge Healthtech Institute (CHI). JCB has arranged a discount for our readers when registering for SelectBio and CHI events, which by the way, have many presenters that are members of our Associate Editors and Editorial Board. Additional information about such opportunities can be found on JCB's news page (bit.ly/1p9EXVd).

As an ongoing partnership/collaboration with the BioPharma Research Council (http://www.biopharmaresearchcouncil.org/), the Editor-in-Chief, Winston Patrick Kuo organized three events around the “microbiome”, two as a webinar series and the other, a conference hosted by the National Cancer Institute. In the first webinar, we were able to utilize our Editorial team to suggest the theme and present in the first session “Introduction to the Microbiome” by Alexander “Sasha” Vlassov, PhD, and as well as other talks from Camilia Martin, MD, MS of the Harvard Medical School, and Floyd Dewhirst, PhD, of The Forsyth Institute. The second webinar was held as a four-session webinar series in April 2015 entitled “Short Course in the McRobiome”. The goal of the short course was to provide insights at an introductory level, yet also a broad overview of the field in the form of an exchange between researchers from academia and industry in the clinical applications of the oral and gut microbiome. We had world renowned speakers including Rob Knight from UCSD, Camilia Martin from the Harvard Medical School, and Romina Goldszmid and K. Leigh Greathouse both from the NIH. They covered topics related to the “system biology approach”, “spatially explicit maps”, “microbiome and cancer therapy” and a “round-up of the latest news”. A meeting dispatch was published online and is available on the *Journal of Circulation Biomarker's* site (http://www.intechopen.com/journals/journal-of-circulating-biomarkers/short-course-in-the-microbiome). The event at the NCI was themed “Altering the Microbiome: Can it Impact Health?”. The purpose of this was to discuss how the host microbiome may be altered and whether such approaches can result in a positive impact on the host. We learned that the preparation process required about six months of coordination. We plan to continue several webinar series on circulating tumour cells and cell-free circulating DNA later this year.

As we strive to publish the best science in all fields of circulating biomarkers, the Editorial team plans to focus on additional topics such as cell-free DNA and disruptive technologies, among others.

JCB accepts original and review articles as well as editorials, perspectives, short research reports, protocols and methods, notes to the Editor, letters to the Editor and meeting dispatch reports. We would like to mention that InTech have decided that JCB will not apply any article processing charges for authors whose research papers have been accepted for publication in Volume 5/2016. Manuscript processing and peer-reviewing for JCB is conducted entirely online. The Editorial Manager facilitates the manuscript processing time, reducing costs and providing a better experience for our authors and reviewers. We have received numerous positive comments on this evolving electronic system, and we are indebted to InTech Open Access Publisher for managing this process for us. For more information, please visit our new manuscript submission system at http://www.editorialmanager.com/exo/default.asp. In addition, we are looking to expedite the acceptance to publishing time for our authors and will request informal opinions from our Editorial Board for borderline cases.

As an ongoing effort, JCB will continue to work on partnering with scientific societies, and academic and industry leaders to advance the field and to allow free access to knowledge in the circulating biomarker space. It's our great pleasure to thank the Editorial team and publishers who make this possible.

